# CDC20 regulates cardiac hypertrophy via targeting LC3-dependent autophagy: Erratum

**DOI:** 10.7150/thno.68367

**Published:** 2021-11-10

**Authors:** Yun-Peng Xie, Song Lai, Qiu-Yue Lin, Xin Xie, Jia-Wei Liao, Hong-Xia Wang, Cui Tian, Hui-Hua Li

**Affiliations:** 1 Department of Cardiology, Institute of Cardiovascular Diseases, First Affiliated Hospital of Dalian Medical University, Dalian 116011, China;; 2 Department of Cardiology, Peking University Third Hospital and Key Laboratory of Cardiovascular Molecular Biology and Regulatory Peptides, Ministry of Health, Key Laboratory of Molecular Cardiovascular Sciences, Ministry of Education and Beijing Key Laboratory of Cardiovasicular Receptors Research, Beijing 100191, China;; 3 Department of Physiology and Pathophysiology, School of Basic Medical Sciences, Capital Medical University, Beijing 100069, China.

The authors regret that the original version of this paper 1 unfortunately contained a labeling error and some incorrect images in Figure 4B. Those are due to typo and improper image selection. This corrigendum does not affect any results or conclusions of the paper. The authors sincerely apologize for any confusion and inconvenience that it may have caused. The correction has now been made online.

## Figures and Tables

**Figure 1:**
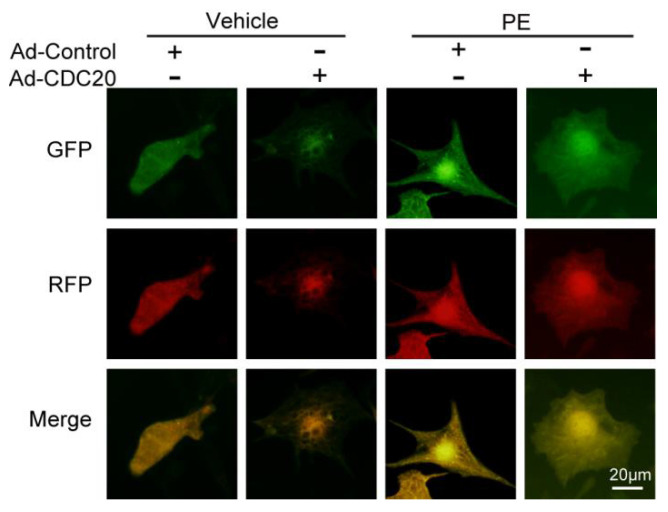
Corrected image for original Figure 4B.

## References

[1] Yun-Peng Xie, Song Lai, Qiu-Yue Lin, Xin Xie, Jia-Wei Liao, Hong-Xia Wang, Cui Tian, Hui-Hua Li (2018). CDC20 regulates cardiac hypertrophy via targeting LC3-dependent autophagy. Theranostics.

